# A highly conserved ABCG transporter mediates root–soil cohesion in Arabidopsis

**DOI:** 10.1093/plphys/kiaf193

**Published:** 2025-05-12

**Authors:** Bethany M Eldridge, Emily R Larson, Lucy Mahony, James Clark, Jumana Akhtar, Clarice Noleto-Dias, Jane L Ward, Claire S Grierson

**Affiliations:** School of Biological Sciences, University of Bristol 24 Tyndall Ave, Bristol BS8 1TQ, UK; School of Biological Sciences, University of Bristol 24 Tyndall Ave, Bristol BS8 1TQ, UK; The Earlham Institute, Norwich NR4 7UZ, UK; School of Biological Sciences, University of Bristol 24 Tyndall Ave, Bristol BS8 1TQ, UK; School of Biological Sciences, University of Bristol 24 Tyndall Ave, Bristol BS8 1TQ, UK; Plant Sciences and the Bioeconomy, Rothamsted Research, West Common, Harpenden. Herts AL5 2JQ, UK; Plant Sciences and the Bioeconomy, Rothamsted Research, West Common, Harpenden. Herts AL5 2JQ, UK; School of Biological Sciences, University of Bristol 24 Tyndall Ave, Bristol BS8 1TQ, UK

## Abstract

Identifying plant molecular mechanisms that mediate root–substrate interactions might offer potential solutions to soil erosion, especially in crop fields, where agricultural practices lead to soil loss. Mutants of the Arabidopsis (*Arabidopsis thaliana*) *ATP-Binding Cassette G 43* (*ABCG43)* transporter gene show enhanced root–substrate cohesion, even though their root micro- and macro-structures are similar to those of wild-type Arabidopsis. We used genetic, biochemical, and functional methods to characterize the substrate-binding effects of changes in *ABCG43* expression, including differences in exudate composition, and phylogenetic analyses to explore the evolutionary history of ABCG43 in land plants. Exudates from roots of the *abcg43* mutant bound more soil and growing medium, and there were significant differences in *abcg43* root exudate composition compared with the wild type. These results suggest that ABCG43 normally functions to mediate root exudates that affect root–substrate cohesion. Phylogenetic analysis showed that ABCG43 is highly conserved in plants, including in agriculturally important crop species. These results provide evidence that ABCG43 is a promising molecular target for developing crop plants with enhanced root–soil cohesion.

## Teaser

The ABCG43 transporter modifies root exudate composition and root–substrate interactions, making it a promising molecular target for crop improvement.

## Introduction

Plant roots protect the soil from erosion but the plant-specific traits driving cohesive interactions between roots and their substrate(s) are unknown (Gyssels et al. 2005; Zhou and Shangguan 2007, 2008; Ola Dodd and Quinton 2015; Burak Quinton and Dodd 2021). Some of these limitations are due to plant biology and soil biology research using very different experimental procedures, tools, and models as well as a lack of integrated and quantitative methods that can measure plant root-dependent contributions to soil properties. Comparative analyses have shown that architectural root traits, such as lateral root length and depth (Ennos 1989; Bailey Currey and Fitter 2002; Burylo et al. 2012), and root hair number, length and density (Akhtar et al. 2018; De Baets et al. 2020), can contribute to the mechanical interactions between plant roots and their environments. Compounds produced by roots (i.e. exudates) can also shape the biotic and abiotic properties of the root–substrate interface (i.e. rhizosphere) and participate in physiochemical and biological interactions (Badri and Vivanco 2009; Baetz and Martinoia 2014; Mommer Kirkegaard and van Ruijven 2016; Oburger and Jones 2018; Sasse Martinoia and Northen 2018). On their own, exudates have soil-binding properties, even in sterile conditions (Akhtar et al. 2018; Galloway et al. 2020, 2022), suggesting that these compounds are not only produced to recruit microbes but also participate directly in plant–soil interactions.

Working models suggest that root exudates are passively and actively released into the rhizosphere (Badri and Vivanco 2009; Weston Ryan and Watt 2012; Baetz and Martinoia 2014; Vives-Peris et al. 2020). Passive release includes the sloughing of root cap cells, diffusion of low molecular weight exudate compounds across cell membranes, and the secretion of high molecular weight compounds via channels and exocytosis; while active release transporter proteins such as ATP-Binding Cassette (ABC) and multidrug and toxic compound extrusion move root exudates across the plasma membrane (Badri and Vivanco 2009; Weston Ryan and Watt 2012; Baetz and Martinoia 2014; Vives-Peris et al. 2020). The ABC transporter family is well conserved in plants, encoding large transmembrane proteins responsible for importing and/or exporting low and high molecular weight substrates involved in a wide variety of physiological processes (Rea 2007; Dhara and Raichaudhuri 2021; Gräfe and Schmitt 2021). The ABC transporters comprise one of the largest gene families in plants, with the ABCG family being the largest and most diverse, containing both full-size pleiotropic drug resistance and half-size White-Brown Complex transporter proteins, of which the latter can dimerise to form functional transporters (McFarlane et al. 2010; Dhara and Raichaudhuri 2021). The G class of this family is significantly expanded and several of its members are linked to the regulation of root–environmental interactions and plant stress responses (Badri et al. 2008, 2009; Eldridge et al. 2020; Dhara and Raichaudhuri 2021; Gräfe and Schmitt 2021; Jarzyniak et al. 2021). For example, the Arabidopsis mutant of *ABCG30* (*pdr2*) has an altered root exudate composition and enhanced root-gel adhesion relative to wild-type seedlings (Badri et al. 2008, 2009; Eldridge et al. 2021). Additionally, the rice *ABCG43* gene can promote heavy metal tolerance when expressed in a yeast homologous system (Oda et al. 2011) and increases cadmium (Cd) accumulation within rice cells, ostensibly through the transport of Cd into the tonoplast for sequestration (Tian et al. 2023). These findings add to a growing body of literature that shows ABCG proteins can mediate root–environment interactions in many plant species via the transport of diverse substrates.

Based on reported contributions of ABCG transporter proteins to root–environmental interactions (Badri et al. 2008, 2009; Tian et al. 2023) and previous evidence that ABCG43 affects root–substrate adhesion in Arabidopsis (Eldridge et al. 2021), we hypothesize that ABCG43 alters exudate composition to mediate root–substrate interactions. Here, we present the evolutionary conservation of ABCG43 in land plants, which predicts that its orthologs in many important crop species share structural and potentially functional similarity. Our comprehensive analysis using the genetic tools available in *Arabidopsis thaliana* examines how AtABCG43 contributes to root–environment interactions to modify root–substrate cohesion, illustrated by enhanced root–substrate adhesive/cohesive properties that correspond with changes in exudate composition between *atabcg43* mutants and wild-type (Col-0). These results highlight the importance of ABCG transporters in regulating root–environmental interactions and provide foundational knowledge of AtABCG43 as a mediator of exudate composition and root–substrate cohesion, making it a promising target for crop improvement.

## Results

### The evolution of ABCG43 across land plants

**Figure 1.**
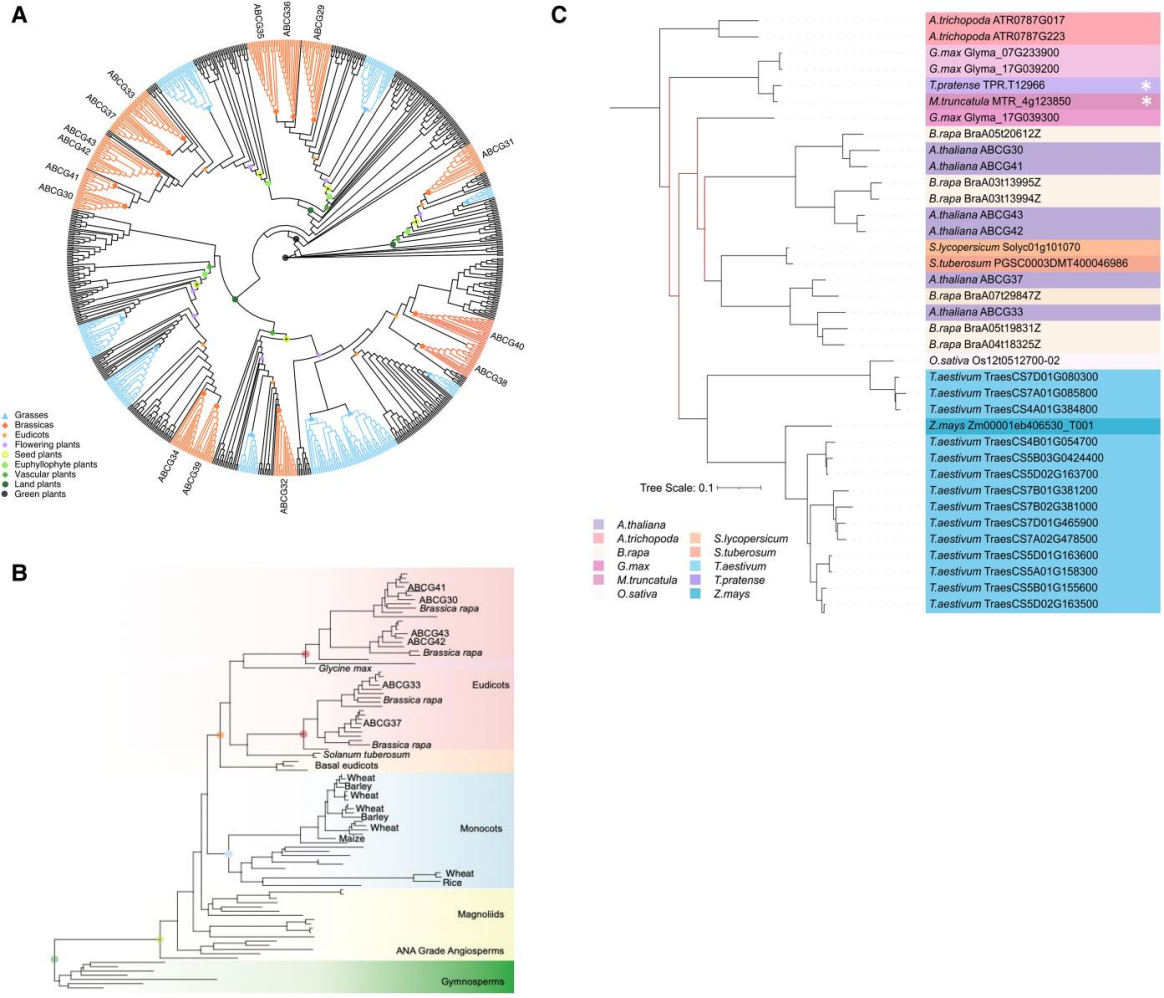
The evolution of the ABCG43/2 gene family across land plants and its orthologs in crop species. **A)** The number of gene copies at key nodes in the tree is shown as colored symbols, with the width proportional to the number of genes. **B)** The resolved subfamily containing ABCG43/2. Clades are colored. Gene copies in *Arabidopsis thaliana* and notable crop species are marked. **C)** The resolved maximum likelihood phylogenetic tree of the ABCG subfamily containing ABCG43. Bootstrap values <100 indicated by red nodes. The tree scale represents number of amino acid substitutions per site. White asterisks indicate cover crop species.

The ABC transporter families are some of the most well conserved protein families found in eukaryotic organisms, with 28 ABCG genes in Arabidopsis (Verrier et al. 2008; Andolfo et al. 2015; Lane et al. 2016). In a screen for Arabidopsis mutants with altered root–substrate adhesion, the *atabcg43* mutant had increased root–substrate adhesion, suggesting that AtABCG43 regulates interactions between plant roots and their growth environments (Eldridge et al. 2020). To place ABCG43 into a larger evolutionary context, we generated a phylogenetic tree sampling genomes across land plants. *ABCG43* belongs to a plant-specific subfamily with a single homolog in the algal species *Chlorokybus* but multiple duplication events resulted in 14 copies in *A. thaliana*, including the transporter genes *AtABCG29* - *34* and *AtABCG37* - *43* (Fig. 1A). These homologs appear to have arisen via duplication events at varying taxonomic scales, resulting in homoeologous relationships among the gene copies within *A. thaliana* and other species. We observed at least 3 copies preserved across land plants, indicating at least 2 land plant-wide duplication events. Subsequent duplications were frequently observed in vascular plants, euphyllophytes, seed plants, and angiosperms. The *ABCG43* genes were part of a Brassicaceae-specific subfamily consisting of *ABCG30, 33, 37, 42,* and *43* (Fig. 1B), with *ABCG42* and *43* arising from a duplication only within *A. thaliana.* All other sampled species within the *Arabidopsis* genus possessed a single, pre-duplicate homolog of *AtABGCG43/2*, while all sampled genomes of *A. thaliana* possessed 2 paralogs. The proximity of the 2 genes along the same chromosome supports a recent tandem duplication event, with only minimal sequence divergence between the 2 paralogous copies. This phylogenetic analysis indicated that ABCG43 is well conserved across land plants, with the potential for a similar function in diverse plant species.

### AtABCG43 homologs present in crop species

Because an AtABCG43 homolog is present across all plant lineages, we asked if ABCG43 is conserved in agriculturally important crop species by generating a gene phylogeny for AtABCG43 homologs in crops that were chosen due to their agricultural importance, well-annotated genomes, use as cover crops (Dapaah and Vyn 1998; Eldridge 2020; van Delden et al. 2021) and in vertical farming (Eldridge 2020; van Delden et al. 2021). We identified at least one ABCG43 homolog in every species included in the analysis (Fig. 1C). Homologous sequences were highly similar to the Arabidopsis ABCG43 protein (E-value < 3.81×10^−148^ for all comparisons, Supplementary Table S1). The phylogeny suggests that the homologs function as ABCG proteins and may have similar functions as those observed in Arabidopsis (Eldridge et al. 2021). Although these findings do not demonstrate that these AtABCG43 homologs transport the same substrates, they are promising molecular target candidates for altering root–soil cohesion in crop plants.

### AtABCG43 is localized to the plasma membrane in Arabidopsis roots

**Figure 2.**
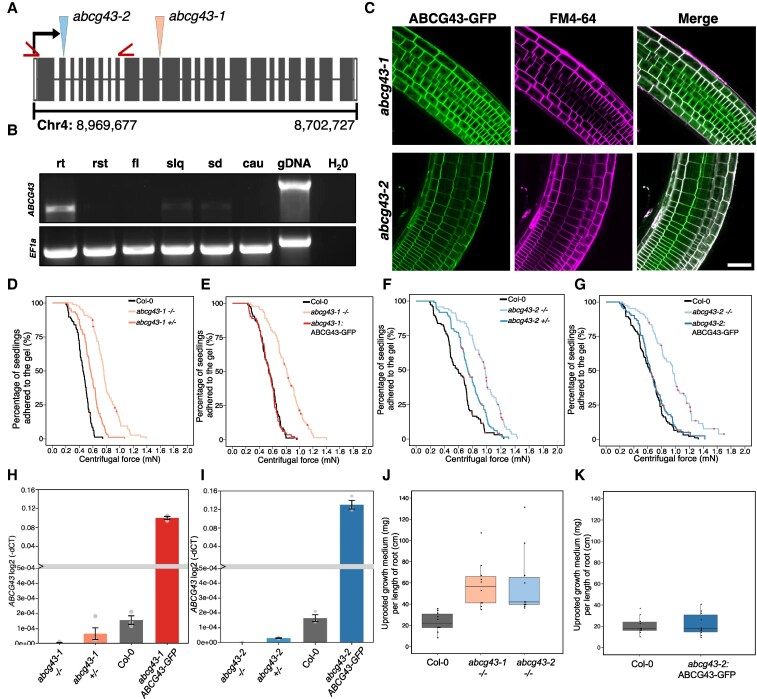
*AtABCG43* expression affects root-substrate cohesion. **A)** T-DNA insert locations in ABCG*43* for each mutant allele. Insertions are indicated by arrowheads. The binding sites of the ABCG*43-*gene-specific primers used in the RT-PCR analysis are indicated by red carrots. **B)** RT-PCR analysis of *AtABCG43* expression in different Col-0 Arabidopsis tissues. Root (rt), rosette leaf (rst), flower (fl), silique (slq), seedling (sd), and cauline leaf (cau)*. EF1-α* was used as an expression control. **C)** The pUBQ10:ABCG43-GFP construct was stably transformed into *abcg43-1* and *abcg43-2* mutant lines. FM4-64 was used as a plasma membrane marker. Scale bar = 50 *µ*m. Survival curves showing the gene dose dependent adhesion phenotypes of (**D–G**) *abcg43-1* and *abcg43-2* homozygous, heterozygous, and complemented lines in comparison to Col-0 controls. Red crosses on the survival curves represent seedlings that remained adhered to the gel after the maximum centrifugal speed (1,611 RPM). Each graph illustrates representative data from at least 2 independent experiments and show a statistically significant difference in adhesion between mutant lines relative to wild type (Cox PH regression; α = 0.01). **H,I)** qRT-PCR analysis of *ABCG43* transcript levels measured by the mean log2 (-dCT) values (+-SE; n = 3) in the homozygous, heterozygous and complemented *abcg43* mutant alleles, and Col-0. The amount of compost attached to uprooted plants of wild type and *abcg43* mutant alleles, showing more compost attached to the roots of the **J)**  *abcg43* mutants and **K)** complemented line compared to wild-type roots after uprooting (*P < 0.001, see Table 2). Data are representative of 2 experiments (*n* = 10–15 plants per genotype).

ABCG transporters primarily localize to the plasma membrane in Arabidopsis and other plant species (McFarlane et al. 2010; Banasiak et al. 2020; Gräfe and Schmitt 2021; Jarzyniak et al. 2021). There are 3 independent homozygous T-DNA insertional mutants that were identified to have similar effects on root–substrate adhesion (Eldridge et al. 2021), of which we chose the first 2 alleles for our studies (Fig. 2A). The gene expression profile showed that *AtABCG43* was expressed mainly in the roots, with no or low expression in other tested tissues (Fig. 2B). Because the endogenous *AtABCG43* was expressed at low levels, we used an expression vector with the constitutive ubiquitin 10 promoter (Grefen et al. 2010) to ensure visualization of the transgene in Arabidopsis. The pUB10:AtABCG43-GFP localized to the plasma membrane of root epidermal cells in both complemented *atabcg43* mutant lines, indicated by its colocalization with the lipid dye FM4-64 (Figs. 2C and Supplementary S1A; Supplementary Table S2; mean Pearson's corelation coefficients for *abcg43-1:ABCG43:GFP*  *=* 0.888 ± 0.023 and *abcg43-1:ABCG43:GFP*  *=* 0.970 ± 0.015; *P* < 0.001 in all cases). While we did observe some difference in fluorescence intensity between the alleles, there was no significant difference in fluorescence between the independent lines of each mutant allele (Supplementary Fig. S1B and C). Interestingly, we also observed that the AtABCG43-GFP fusion protein in puncta within root cells, especially at the root tip where cells are actively expanding. These puncta did not co-localize with those labelled with FM4-64, which may suggest that AtABGC43 is secreted *de novo* to the plasma membrane and not endocytosed or recycled (Supplementary Fig. S1D).

### AtABCG43 root–substrate adhesion effects are gene-dose dependent

To examine the range of effect AtABCG43 has on root adhesion, we back-crossed the *atabcg43* mutants to wild type (Col-0) to produce heterozygous *atabcg43+/−* lines in both mutant allele backgrounds. The homozygous *abcg43* mutants (*abcg43−/−*), heterozygous *abcg43* mutants (*atabcg43+/−*), and the *atabcg43-1* and *atabcg43-2* mutants complemented with the pUB10:AtABCG43-GFP construct showed no differences in root hair growth and development when compared with Col-0 (Supplementary Figs. S2 and S3; Supplementary Table S3). We tested the root–substrate adhesion of these transgenic lines compared to Col-0 using a centrifuge-based adhesion assay (Eldridge et al. 2021). While the roots of *abcg43−/−* seedlings were more adhesive compared to Col-0, the *atabcg43+/−* seedlings only partially rescued this mutant phenotype and those expressing AtABCG43-GFP were not significantly different from Col-0 (Fig. 2D–G; Table 1). Taken together, these results are consistent with ABCG43 contributing to the root adhesion in a way that does not affect the physical microstructures of the root.

**Table 1.** Cox PH regression models comparing the root-gel detachment of atabcg43-1 mutant, backcrossed, and complemented lines relative to wild type (Col-0)

Line	Wald test (*z*-score) and corresponding *P* value		Hazard ratio (95% CI)
*atabcg43-1 −/−*	*z* = −11.99 *P* < 0.001	***	0.09 (0.06, 0.14)
*atabcg43-1 +/−*	*z* = −6.62 *P* < 0.001	***	0.33 (0.24, 0.50)
*atabcg43-1: AtABCG43-GFP*	z = 0.16 *P* = 0.861	**ns**	1.03 (0.77, 1.40)
*atabcg43-2 −/−*	z = −8.40 *P* < 0.001	***	0.26 (0.19, 0.40)
*atabcg43-2 +/−*	z = −3.91 *P* < 0.001	***	0.57 (0.43, 0.80)
*atabcg43-2: AtABCG43-GFP*	z = −1.10 *P* = 0.292	**ns**	0.85 (0.63, 1.15)

^***^Indicates a statistically significant difference ≤ 0.001 relative to Col-0. “ns” indicates no significant difference relative to Col-0.

The gene transcript levels in the *atabcg43+/−* and AtABCG43-GFP complemented lines were measured by qRT-PCR, which showed that the *atabcg43+/−* lines had approximately half as much expression of *ABCG43* as Col-0 (Fig. 2H, I, Table 2), indicating that *AtABCG43* gene expression has a dose-dependent effect on root–substrate adhesion properties. The complemented lines showed some variability in *AtABCG43* expression, which was probably due to the ubiquitin promoter used to drive the transgene. However, in all cases, the gene expression results were at least as high as wild type, consistent with the phenotypic complementation observed.

**Table 2.** AtABCG43 expression in transgenic lines relative to wild type (col-0)

Line	Mean *ABCG43* expression (log2 (−dCT) ± standard error)	Fold difference in *ABCG43* expression (log2 (−dCT))	
Col-0	1.56×10^−4^ (±7.81×10^−5^)	*n* *=* 3	−
*atabcg43-1−/−*	4.99×10^−6^ (±2.22×10^−6^)	0.03-fold lower than Col-0 *n* *=* 4	t = −6.31 *P* < 0.01** d.f. = 4
*atabcg43-1+/−*	2.73×10^−5^ (±1.37×10^−5^)	0.18-fold lower than Col-0 *n* *=* 4	t = −4.52 *P* < 0.05* d.f. = 4
*atabcg43-1:*ABCG43-GFP	0.10 (±0.02)	638.50-fold higher than Col-0 *n* *=* 3	t = 32.77 *P* < 0.001*** d.f. = 4
Col-0	1.66×10^−4^ (±8.33×10^−5^)	*n* *=* 3	-
*atabcg43-2−/−*	4.92×10^−7^ (±2.56×10^−7^)	3.00×10^−6^-fold lower than Col-0 *n* *=* 4	t = −7.31 *P* < 0.01** d.f. = 4
*atabcg43-2+/−*	3.13×10^−5^ (±1.37×10^−5^)	0.19-fold lower than Col-0 *n* *=* 4	t = −5.91 *P* < 0.01** d.f. = 4
*atabcg43-2:*ABCG43-GFP	0.13 (±0.04)	781.20-fold higher than Col-0 *n* *=* 3	t = 13.91 *P* < 0.001*** d.f. = 4

Mean difference and output of linear model (Student's *t*-test) for each candidate line relative to Col-0. ***Statistical significance ≤ 0.001, **Statistical significance ≤ 0.01, and *Statistical significance ≤ 0.05.

### AtABCG43 affects root–substrate adhesion in mature plants grown in growth medium

To test whether AtABCG43 contributes to root cohesive interactions in mature plants grown in a complex growth substrate, Col-0 and *atabcg43* mutant plants were grown in growth medium to the vegetative stage just before flowering and uprooted using a tensile testing machine. We then quantified how much growth medium was associated with a standardized length of root (De Baets et al. 2020). The *atabcg43* mutant lines had approximately 2.2–2.6 times more uprooted growth medium per cm length of root than Col-0 (Fig. 2J; Table 3). We also compared the uprooted growth medium between Col-0 and the AtABCG43-GFP complemented lines and found no significant difference in uprooted growth medium per length of root between the lines (Fig. 2K; Table 3). Differences in root length density and uprooted root length between these lines could contribute to root–substrate interactions; therefore, we calculated the total root length density (RLD) and total uprooted root length for the plants used in the uprooting experiments. There was no difference in these parameters between the Col-0 and transgenic lines (Supplementary Fig. S4 and Supplementary Tables S4 and S5), which was consistent with those from the centrifuge assay and indicated that AtABCG43 contributes to root–substrate interactions at different stages of plant development and across different growth conditions.

**Table 3.** AtABCG43 expression alters root-growth medium cohesion in uprooted plants compared with col-0

Line	Mean uprooted growth medium per cm of root (mg ± standard error)	Difference between uprooted growth medium (mg)	
Col-0	23.13 (±0.212)	-	-
*atabcg43-1*	58.22 (±0.194)	2.52 times more *n* = 10	t = 3.42 *P* < 0.001*** d.f. = 18
*atabcg43-2*	59.84 (±0.163)	2.59 times more *n* = 10	t = 3.53 *P* < 0.001*** d.f. = 18
Col-0	20.76 (±2.567)	-	-
*atabcg43-2: AtABCG43-GFP*	21.37 (±1.570)	No difference *n* = 10	t = 0.33 *P* > 0.05*^ns^* *d.f.* = 18

Mean growth medium attached to uprooted Arabidopsis roots (±standard error). Mean difference and output of univariate linear model (*t*-test) for each candidate line relative to Col-0. Representative results from 2 experiments (*n* = 10 for each genotype). ***Statistical significance ≤ 0.001 and ns, no statistical significance.

### Exudate composition is altered in *atabcg43* mutants

**Figure 3.**
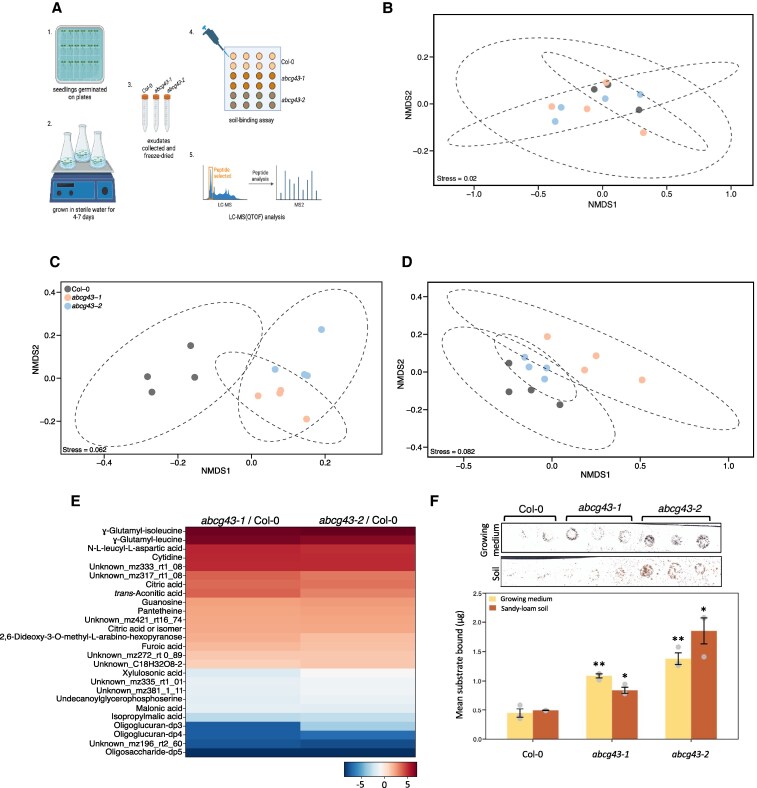
*Abcg43* mutant exudates enhance root-substrate cohesion. **A)** Schematic of exudate collection from Arabidopsis seedlings. (i) Seedlings are germinated on 0.5X MS medium for 4 d. (ii) Seedlings are transferred into sterile water in flasks and grown with agitation for 3 d. (iii) The growth liquid is collected and freeze dried for use in (iv) soil binding and (v) metabolite analyses. Schematic made in BioRender. **B)**  ^1^H-NMR data from exudates collected from Col-0 and *abcg43* mutant seedlings. Non-metric multidimensional scaling (NMDS) analysis of LC-QTOF in **C)** negative and **D)** positive ion modes. **E)** Heatmap of LC-orbitrap results comparing metabolites present in Col-0 and *abcg43* mutant exudates. **F)** Representative scanned nitrocellulose sheet showing the growing medium and soil bound by Col-0 and *abcg43-1* and *abcg43-2* soluble root exudates. Exudates were added to nitrocellulose membranes as 5 μL dots containing 50 μg soluble root exudates. The sieved growing medium and soil that bound to the exudate samples were quantified by use of a calibration curve. Each data point is a mean of 3 technical and biological replicates; error bars indicate the standard error. ** = *P* < 0.01 and * = *P* < 0.05 when compared with Col-0 in the same growth conditions.

We next asked if there were changes in the exudate composition and adhesion properties between the *abcg43* mutants and Col-0. We collected exudates from 7-day-old seedlings using a protocol that allowed us to assess soil binding properties and composition using untargeted metabolomics (Fig. 3A). We first examined the Col-0 and *abcg43* mutant exudates using ^1^H-NMR spectroscopy. Here, the major signals related to fructose dominated the spectra and a comparison of fructose levels across the samples suggested that levels were higher in the *abcg43* mutants compared to Col-0, although sample-to-sample variability within the biological replicates for this metabolite resulted in large error bars and the significance of these differences could not be validated (Supplementary Fig. S5E). This variability was further reflected in the NMDS of the ^1^H-NMR data, which showed no difference among the *abcg43* mutants and Col-0 (Fig. 3B). Liquid chromatography-mass spectrophotometry (LCMS) analysis in both negative and positive ionization modes generated a list of metabolite features in both Col-0 and the *abcg43* mutant alleles. These features included organic acids, flavonols, phenylpropanoids, nucleosides, amino acids, deoxynucleosides, indols, fatty acid derivatives, coumarins, megastigmanes, glucosinolate degradation products, and dipeptides. Non-metric multidimensional scaling (NMDS) of the LC-MS data visualized the differences in exudate composition between the *abcg43* mutants and Col-0, which indicated clear associations between the mutant alleles in the data collected in negative ionization mode but fewer differences in metabolites observed in positive mode (Fig. 3C, D). Of the 249 features identified in the negative ionization, 25 features were differentially regulated in both *abcg43* mutant alleles compared to Col-0 (Fig. 3E). Among those that were upregulated, we highlight the dipeptides γ-glutamyl-isoleucine, γ-glutamyl-leucine, N-L-leucyl-L-aspartic acid; the nucleosides cytidine and guanosine; and the organic acids citric, isocritic, aconitic and furoic acids. Their structures were confirmed by MS/MS whenever reference standards were not available (Supplementary Fig. S5A-D). These results showed that the *abcg43* mutant alleles differentially express exudate compounds compared with Col-0, which is consistent with the diverse changes observed in exudate composition of other ABCG transporters like ABCG30 (PDR2) and ABCG34 (PDR6) (Badri et al. 2008).

Based on the compositional changes in the *abcg43* mutant root exudates when compared to Col-0, we hypothesized that AtABCG43 affects root–substrate interactions by altering the binding capacity of exudates. We tested this hypothesis using a soil-binding assay (Akhtar et al. 2018). The exudates collected from the *atabcg43* mutants were able to bind 2.43–3.08 times more growth medium and 1.71–2.20 times more soil than Col-0 (Fig. 3F; Table 4), which was consistent with both the centrifuge assay and uprooting experiment results (Fig. 2D–G, J, K). Altogether, these findings indicated that AtABCG43 affects root–substrate interactions and that it most likely does so by mediating root exudate composition.

**Table 4.** The atabcg43 mutant exudates bind more growth-medium and soil than col-0 exudates

Substrate	Line	Mean substrate bound (μg ± standard error)	Difference between substrate bound (μg)	
Growing medium	Col-0	0.49 (±0.073)	-	-
	*atabcg43-1*	1.09 (±0.349)	2.43 times more than Col-0 *n* *=* 3	t = −7.94 *P* < 0.01** d.f. = 2.88
	*atabcg43-2*	1.38 (±0.100)	3.08 times more than Col-0 *n* *=* 3	t = −7.53 *P* < 0.01** d.f. = 3.64
Sandy-loam soil	Col-0	0.49 (±0.004)	-	-
	*atabcg43-1*	0.84 (±0.052)	1.71 times more than Col-0 *n* *=* 3	t = −6.779 *P* < 0.05* d.f. = 2.02
	*atabcg43-2*	1.85 (±0.221)	2.20 times more than Col-0 *n* *=* 3	t = −6.156 *P* < 0.05* d.f. = 2.00

Mean difference and output of linear model (Welch's t-test) for each candidate line relative to Col-0. **Statistical significance ≤ 0.01 and *Statistical significance ≤ 0.05.

Collecting and analysing root exudates with the soil binding assay and metabolomics showed that the loss of ABCG43 function in plant roots alters the chemical root exudate composition and affects root–substrate interactions. These results also support the use of metabolomics and soil-binding methods for quantifying plant-specific contributions to root–substrate interactions. Together with the results showing that ABCG43 is a highly conserved protein in land plants and that the loss of ABCG43 does not affect root micro- or macro-structures, our overall findings suggest that ABCG43 is a promising target for enhancing root–soil interactions without affecting plant development.

## Discussion

Identifying plant traits that protect against soil erosion is vital for developing sustainable control measures that protect against erosive forces and enhance crop productivity. We present ABCG43 as a plant transporter protein that mediates cohesive interactions between roots and their environment.

The size of the ABCG43 gene family is reflected in the history of duplication and loss across land plants, with an ancient origin at least within the green algae. Based on their functional annotation, the diversification of the gene family has been accompanied by divergence in gene function and localization across various plant tissues (Ashraf et al. 2021; Cho et al. 2021; Eldridge et al. 2021). Despite the deep origins of the family and its functional divergence, ABCG43 and its paralog ABCG42 only arose very recently, likely via a tandem duplication specifically within *A. thaliana,* with only a single pre-duplication gene copy present in all other *Arabidopsis* species sampled. The confinement of ABCG43/2 to a single genome and their highly conserved sequence suggests minimal functional divergence; yet, the retention of both copies indicates the potential for subfunctionalization. However, the paralogy between the 2 genes is only relevant within *A. thaliana*, since most other species possess only a single homoeologous copy or have undergone subsequent independent duplication events. The large family of ABCGs with deep origins evidences the potential for conserved function across land plants and that through repeated duplications, there is the potential for diversification of function. This opens more questions for future research, since many of these genes could also be involved in root–soil interactions, potentially through mediating exudate composition. These evolutionary analyses identified conserved regions of the genes and proteins that may support conserved function with respect to mediating root–substrate interactions. Further research will be needed to evaluate any functional relationship between *AtABCG43* and *AtABCG42*, as well as explore the evolutionary or ecological consequences of this duplication in *A. thaliana.* Within the *ABCG43/2* subfamily, *ABCG37* and *ABCG33* possess root adhesion or nutrient uptake phenotypes, supporting the hypothesis that this subfamily is associated with root–substrate interactions (Ashraf et al. 2021; Eldridge et al. 2021). Further research will be needed to understand the functional conservation of ABCG43 among land plants and the relevance of how plant roots can use ABCG transporter function to mediate root–environmental interactions.

ABCG transporters are large genes that encode complex transmembrane proteins and experimental data on their function is limited. The use of genetic mutants has provided some physiological evidence of their crucial functions in hormone transport, cuticle formation, pathogen resistance, pollen wall formation, microbial interactions, and heavy metal resistance (McFarlane et al. 2010; Banasiak et al. 2020; Dhara and Raichaudhuri 2021; Jarzyniak et al. 2021). The ABCG43/2 family has homologs in the grasses (Fig. 1A, B), and so we predict they could have a similar role in mediating root–environment interactions, albeit with potentially different substrates. Crucially, a homoelogous copy of ABCG43 is present in all major crop species that all have clear transmembrane domains (TMDs) and nuclear binding domains (NBDs) that are indicative of ABC transporters, as well as high sequence similarity to the Arabidopsis ABCG43 protein. While the ABCG family shows broad functional divergence (Verrier et al. 2008; Borghi et al. 2015; Do Martinoia and Lee 2018) and conserved function can only be determined experimentally, this bioinformatic analysis of the ABCG43/2 sequence similarity among land plants provides support for potentially conserved functions with respect to mediating root–substrate interactions (Fig. 1B, C). This hypothesis is consistent with studies that showed that ABCG transporters have multiple substrates that can alter exudate composition and are important for plant–environment interactions (Badri et al. 2008, 2009). These homoeologs are potential target genes for manipulating root–soil interactions in crop plants to bioengineer the root–soil interface and rhizosphere, and it would be worthwhile testing whether they can benefit applications such as restoring degraded soils or increasing soil organic carbon storage for enhanced carbon sequestration (Ledo et al. 2020; Eckardt et al. 2023).

The Arabidopsis *abcg43* mutant was initially identified in a screen for mutants with altered root–substrate interactions (Eldridge et al. 2021), suggesting AtABCG43 regulates root adhesive properties. AtABCG43-GFP localization to the plasma membranes of the Arabidopsis root (Fig. 2C) is consistent with previous reports for AtABCG proteins (McFarlane et al. 2010; Fourcroy et al. 2014; Ashraf et al. 2021). Endogenous expression of At*ABCG43* was very low in Col-0 Arabidopsis and mainly expressed in the roots (Fig. 2B), suggesting that this gene may be tightly regulated or expressed in particular regions of the root. The partial rescue of the *atabcg43−/−* mutant phenotype in the *abcg43+/−* heterozygous lines further indicates that even though it is expressed at low levels, *AtABCG43* has physiological effects on root–substrate interactions (Fig. 2D-G). Gene dosage effects can be linked to gene copy number as well as gene regulatory factors (Birchler and Veitia 2014; Bastiaanse et al. 2019; Shi et al. 2020); therefore, *AtABCG43* expression and localization might be tightly regulated to mediate root–substrate interactions in plants. Other ABCG transporters are involved in biotic and abiotic responses (Kuromori et al. 2010; Fourcroy et al. 2014; Fu et al. 2019; Jarzyniak et al. 2021), so the effects of AtABCG43 function may be linked to environmental conditions. This provides evidence of a gene-dose effect on AtABCG transporter function and highlights its importance in root–environment interactions.

The balance between a plant root's ability to move through and adhere to their environment is important to support root growth while still maintaining anchorage within the surrounding soil. The *abcg43* phenotype suggests that the loss of AtABCG43 increases root–substrate binding properties, perhaps because AtABCG43 function normally reduces or mediates root–substrate cohesion. We hypothesize that AtABCG43 function helps to mediate root–substrate adhesive properties and is a potential genetic target for developing plants that have binding properties suitable for their particular growing conditions.

Plant exudates are also thought to contribute to changes in microbe population and activity, which can alter soil properties (Rillig and Mummey 2006; Carvalhais et al. 2015). Leucine and isoleucine-containing dipeptides and organic acids of the TCA cycle were upregulated in the *atabcg43* exudates. The role of these metabolites in plant physiology is usually related to plant–microbe interactions in the rhizosphere and to defence properties (Strehmel et al. 2017); therefore, AtABCG43 may also function to mediate exudate composition to reduce or mitigate plant–microbe interactions (Strehmel et al. 2017). However, the plants used in the centrifuge assay were grown in sterile conditions, highlighting the adhesive effects plant roots have on their own and that secreted exudates can bind to their substrates without other environmental factors, such as soil composition and microbes (Eldridge et al. 2021). In combination with the centrifuge-based assay, we developed an uprooting assay that showed the *abcg43* mutant bound more growing medium when uprooted than the Col-0 or complemented lines (Fig. 2J, K; Table 3). These assays showed that there were increased root–substrate interactions in *atabcg43* mutant seedlings grown on agar plates and mature plants grown in a compost-based growing medium, demonstrating plant-dependent effects mediated by AtABCG43 contribute to root–substrate adhesion and binding in both sterile and non-sterile growth conditions (Fig. 2). We evaluated the root architecture and physiology of the *abcg43* mutants compared to Col-0 and found no differences in root structure, length, or root hair distribution (Supplementary Fig. S2A; Supplementary Tables S3 and S4), suggesting that ABCG43 mediates root–substrate interactions in ways that are not associated with physical root traits. We believe that these findings suggest ABCG43 mediates these interactions via changes in exudate composition, consistent with what has been reported in other ABCG transporter mutants (Badri et al. 2008).

Our analysis of exudates collected from hydroponically grown seedlings confirmed that the composition of the *atabcg43* mutant soluble exudates was different from that of Col-0 and corresponded with more compost/soil particles adhering to *atabcg43* exudates than those of Col-0 (Fig. 3F; Table 4). While analysis of exudate composition showed that fructose was potentially more abundant in the *abcg43* mutant than in Col-0, although there was too much variation within the tested samples to show a statistical difference between the mutant and Col-0 lines (Supplementary Fig. S5E). We did not find that fructose alone could bind soil, suggesting that the compounds that directly contribute to soil binding do so within the context of the other compositional elements of the root exudates. The results of the exudate composition and soil binding analyses indicate that the overall composition of plant root exudates can affect soil binding and root–substrate cohesion, and that these effects are most likely not due to the sole function of one specific exudate compound.

The centrifuge-based adhesion assay and uprooting experiments used seedlings and plant roots directly sown onto growth substrates. It is possible that polymers and other molecules within the plant root exudates contributed to the binding effects we observed in plant roots (Fig. 2D–G, J, K). In the soil-binding assay, which used filtered samples collected from seedlings grown hydroponically, we also measured increased soil binding with *abcg43* exudates than with Col-0 exudates (Fig. 3F and Table 4). Thus, increased binding/adhesion in the *abcg43* mutant was measured across different experimental conditions and suggests that the changes we reported in the metabolomics analyses of the *abcg43* mutant exudates most likely contributed to the enhanced soil binding we measured.

The difficulty in identifying specific regulators of exudate composition has been reported, with mutants in single exudate proteins altering the overall exudate composition, rather than just one exudate component (Badri et al. 2008). Identifying the specific substrates of AtABCG transporters is also difficult because of the number of transporter proteins in each family and their ability to have multiple substrates. Additionally, we cannot rule out that the exudate composition in the *atabcg43* mutants could have affected microbial changes to the rhizosphere during our uprooting and soil-binding assays. We hypothesize that the ABCG43-dependent contributions to plant root–substrate binding could be direct via the transport of molecules that participate in root–soil cohesion, or indirect via interactions or effects on other exudate and environmental components. While additional studies will be needed to explore how different types of exudate molecules are involved in plant root–substrate interactions, we believe that the results from our use of different types of genetic, molecular, biochemical, and mechanical experiments demonstrate that AtABCG43 modifies root–environment interactions and that it is a promising candidate for exploring how plant roots attach to their environment.

This study applied diverse and quantifiable approaches to examine root–environment interactions of the previously uncharacterized ABCG43 transporter, which is deeply conserved in land plants and functions to mediate plant root–substrate interactions in Arabidopsis. Understanding the genetic effects of plants on their environment has applications in crop breeding and provides a platform for developing better methods for evaluating direct and indirect interactions between plants and their environments.

## Materials and methods

### Plant lines and growth conditions

Three independent *abcg43* mutant alleles were previously identified (Eldridge et al. 2021). We used *abcg43-1* (N75206), *abcg43-2* (SALK_201207C), and Columbia-0 wild type (Col-0) for all experiments in this report. For sterile culture, seed was sterilized in 20% bleach and stratified at 4 °C for 48 h. Seeds were sown onto solid medium (0.5X MS, 1% sucrose, 1% agar, pH 5.7) in Petri plates that were sealed with parafilm and oriented vertically in long-day light conditions (21–22 °C; 16 h light/8 h dark; 120–145 *µ*mol m^−2^ s^−1^ light; 60% relative humidity). Seedlings (5–7 d-old) were also transplanted onto growing medium (3:1 Levington F3 compost:J Arthur Bowers horticultural silver sand) for uprooting experiments.

### Backcrosses and genetics analysis

The *abcg43*+/− lines were produced by traditional crossing. Col-0 pollen was used to fertilize *abcg43* flowers and the siliques that developed from one plant were pooled together. Six individual plants from each mutant allele were used for the crosses, providing 6 independent backcrossed lines. The backcrossed lines were genotyped as previously described (Eldridge et al. 2021).

### Root hair analysis

Six-day old seedlings grown on 0.5X MS, 1% sucrose, and 1% agar plates were imaged with a Leica MZ FLIII fluorescence microscope with dark-field lighting. Images were captured on a Nikon D50 camera with a polarizing filter using the SPOT image capture software (SPOT IMAGING) or on an Olympus DP74 CMOS color cooled camera using the Cell Sens Standard V2 advanced Imaging Software (Olympus). Images from 15 to 20 seedlings were used to measure root hair length and density. From each seedling, the lengths of 20 root hairs were measured *ad hoc* with Fiji, version 1.53c (Schindelin et al. 2012), and the Bio-Formats Importer plugin. At least 2 experimental repeats were conducted for each candidate line.

### Centrifuge-based root adhesion assay

The centrifuge-based root adhesion assay was performed as previously described (De Baets et al. 2020; Eldridge et al. 2021). Briefly, Arabidopsis seedlings were sterilized and sown onto 0.5X MS, 1% sucrose, 1% agar medium in 90 mm Petri dishes. The plates were sealed with parafilm and grown vertically in long-day light conditions for 5–6 d. Then, the plates were subjected to increasing g-force using a centrifuge. Several variables such as plate, seedling placement, and shoot weight of each seedling were used in the centrifugal force calculations as previously described (Eldridge et al. 2021). Two independent experiments that included over 70 individual seedlings were conducted and representative data are presented.

### Uprooting assay

The plant uprooting protocol was adapted from a previous report (De Baets et al. 2020). Polytetrafluroethylene-coated aluminium washers with garden wire attached at 4 points along the diameter of the washer were placed on the surface of 600 mL loosely packed growing medium in 375 cm^3^ pots. Seed was sown onto the growing medium such that the aerial tissue could grow through the centre hole of the washer. After 3–4 wks, growth in long-day conditions, each pot was placed in 3 cm water to allow moisture equilibration for 12–16 h. Then, plants were uprooted from pots using and tensile testing machine (Instron 3343) with a 100 Newton load cell at a constant rate of 5 mm min^−1^. At least 15 plants were uprooted per genotype in each experiment. Each experiment was conducted twice. The growing medium attached to the uprooted roots was carefully washed off into a Petri dish and dried completely in a 40 °C oven before the soil weight was recorded. The average root length density (RLD) was calculated from roots collected from 3 to 5 pots of each genotype as previously described (De Baets et al. 2020). Briefly, the uprooted roots were placed on 1% charcoal agar plates and imaged to measure root length for each uprooted plant using Fiji. These roots were then collected, completely dried at 40 °C and the dry weight was recorded.

### RT-PCR and RT-qPCR conditions and analysis

#### RNA extraction and cDNA synthesis

Total RNA was extracted from pools of 200 5-d-old seedlings using the Spectrum Plant Total RNA kit (Sigma-Aldrich) following the manufacturer's protocol. One milligram total RNA was used for single-stranded cDNA synthesis using the Applied Biosystems High-Capacity cDNA Reverse Transcription kit (Thermo Fisher Scientific) following the manufacturer's protocol.

#### RT-PCR

*AtABCG43-*gene specific primers (Supplementary Table s6) were used for RT-PCR with cDNA synthesised from total RNA used as a template. *EF1-α* was used as a positive expression control, while a no-template reaction was used as a negative control. *AtABCG43* RT-PCR product sizes are 879 bp for cDNA and 1464 bp for gDNA. *EF-1α* product sizes were 556 bp for cDNA and 659 bp for gDNA. For each line, RT-PCR was conducted on 3 independent cDNA samples. For the gDNA control, a pooled sample of Col-0 gDNA was used.

#### qRT-PCR protocol and analysis

Bespoke *Arabidopsis thaliana* gene probes with Black Hole Quenchers were designed (Sigma-Aldrich) for use in a 2-step, qRT-PCR TaqMan assay on the Mx5005P Agilent thermal cycler with the FAM, ROX, HEX, and Cy5 filter sets. Primer sequences are provided in Supplementary Table S6.

### Cloning the *AtABCG43* gene

#### Yeast homologous recombination and cloning of the AtABCG43-GFP construct

The *Saccharomyces cerevisiae* strain YPH 500 was used for yeast homologous recombination to construct the pCAMBIAY1300 and pCAMBIAY1300-ABCG43 plasmids. Liquid *S. cerevisiae* cultures were made in YDSM/YEPD media and incubated at 28 °C for 48–72 h.

Yeast homologous recombination was used to construct the pCAMBIAY1300-ABCG43 plasmid. The plasmid was linearized with HindIII and EcoRI and 4 overlapping *AtABCG43* PCR fragments were designed to allow for homologous sequences to recombine into pCAMBIAY1300 in *S. cerevisiae* (Supplementary Table S7). The pCAMBIAY1300-AtABCG43 plasmid was extracted using the Zymoprep Yeast Miniprep II Kit (Zymo Research) according to the manufacturer's protocol and propagated in TOP10 *Escherichia coli* cells. The *AtABCG43* coding region was PCR-amplified from the pCAMBIAY1300-ABCG43 plasmid and inserted into the pDONR207 entry vector (Invitrogen) using the Gateway BP Clonase II enzyme mix (Invitrogen) following the manufacturer's instructions, and then inserted into the pUBC-GFP destination vector (Grefen et al. 2010) using the Gateway LR Clonase II enzyme mix (Invitrogen). Primers used for Gateway cloning are provided in Supplementary Table S6.

### Plant transformation

Arabidopsis stable lines were produced using the agrobacteria floral dip method (Clough and Bent 1998). Positive transformants in the T_1_ generation were selected on 0.5X MS plates supplemented with 10 µg/mL glufosinate (Sigma-Aldrich). Homozygous lines were identified in the T_3_ generation and used for experiments.

### Microscopy and protein localisation

Wild-type and transgenic Arabidopsis lines were grown for 4–5 d on 0.5X MS, 1% sucrose, 1% agar medium (pH 5.7) in long-day light conditions. Seedlings were mounted in water on glass slides and imaged on a Leica SP8 AOBS confocal laser scanning microscope attached to a Leica DM I8 inverted epifluorescence microscope using a 40× oil-immersion objective with a numerical aperture of 1.3. The fluorophores were excited with a 65 mW argon laser at 488 nm and emission signals were collected at 509–515 nm for GFP and 700–710 nm for FM4–64 using HyD detectors. For each experiment, 6–10 seedlings were imaged from each line using the same settings. Independent experiments were conducted at least 3 times.

### Root exudate collection and analysis

#### Plant growth and exudate collection

Approximately 200 Arabidopsis seedlings were sterilized and then germinated on 0.5X MS, 1% sucrose, 1% agar medium (pH 5.7) in long-day light conditions for 5 d before being transferred to 10 mL sterile, deionised water in 50 mL Erlenmeyer flasks and grown for an additional 4 d in long-day light conditions with constant shaking. The growth solutions were collected and passed through a 0.24-μm filter to remove cellular debris and lyophilized for further analysis. This material was used as exudate samples for the soil binding and metabolomics experiments.

#### Extraction procedure

The lyophilized root exudates were dissolved in H_2_O:MeOH (1 mL, 80:20 v/v), vortexed for 30 s, and then centrifuged at 13,200 rpm for 2 min. For LCMS analysis (QToF and Orbitrap), 250 µL of each sample were transferred to an autosampler glass vial. For NMR analysis, 650 µL of each sample were transferred to a new vial and the solvent was evaporated using a Speedvac concentrator (Genevac, Suffolk, United Kingdom) for 2.5 h. The pellet was reconstituted in 650 µL 80:20 D_2_O:MeOD containing 0.01% d4− trimethylsilylpropionate (TSP). Samples were transferred to 5 mm NMR tubes.

#### Liquid chromatography–mass spectrometry (orbitrap)

LC-Orbitrap were recorded on an LTQ-Orbitrap Elite mass spectrometer (Thermo Fisher Scientific) coupled to an Dionex UltiMate 3000 RS UHPLC system as previously described (Harrison et al. 2024). The LC-Orbitrap data were processed in Compound Discoverer 3.3 SP2 (Thermo Fisher Scientific) using the “Untargeted Metabolomics Workflow”. LC-MS (Orbitrap) in negative mode identified 249 features after data mining.

#### Liquid chromatography–mass spectrometry (QTOF)

LC-QTOF was recorded on an Agilent 6546 Mass Spectromer equipped with a Dual AJS electrospray ion source. The mass spectrometer was coupled to an Agilent 1290 Infinity II LC system, equipped with a DAD photodiode array detector G7117A. Chromatographic separation and mass spectra were collected as previously described (Harrison et al. 2024). LC-QTOF data were processed in MassHunter Profinder 10.0 (Agilent) using the Batch Recursive Feature Extraction method. Positive and negative ion mode datasets were processed independently. Datasets were mined manually by deleting duplicated peaks (isotope peaks and fragment products) and features that were also present in the blanks (contaminants/impurities). After data mining, negative and positive ion mode LC-MS (QTOF) data were comprised of 321 and 250 features, respectively.

#### ^1^H Nuclear magnetic resonance spectroscopy (NMR) 

^1^H-NMR spectra were acquired under automation at 300°K using an Avance Neo Spectrometer (BrukerBiospin) operating at 600.0528 mHz and equipped with a cryoplatform and a 5 mm triple inverse cryoprobe. Spectra were collected and converted to ASCII files containing integrated regions or “buckets” of 0.01 ppm equal width as previously described (Harrison et al. 2024).

#### Metabolites identification

Peak annotation was made by comparison to known standards run under the same conditions where possible. Putative identifications were made via comparison to the literature of known metabolites identified in Arabidopsis root exudates and more generally in plants, with a molecular formula search in the Reaxys database.

### Soil adhesion assay

The soluble exudates from 200 Arabidopsis seedlings of Col-0, *abcg43-1* and *abcg43-2* were collected as described above. Control polymers and exudates were dissolved in deionised water to concentrations: 10 μg/μL, 2 μg/μL, and 0.4 μg/μL. Aliquots of 5 μL of these samples were spotted onto nitrocellulose sheets (GE Healthcare, Amersham Protran 0.45 NC) and left to air dry for 2 h before the nitrocellulose sheets were processed with sieved (<500 μm) growing medium or sandy loam soil as described previously (Akhtar et al. 2018). Dilutions of 10 μg/μL, 2 μg/μL, and 0.4 μg/μL Gum Tragacanth (Sigma, 9000-65-1) and xanthan gum (Sigma, G1253) were used as positive controls. The nitrocellulose sheets were weighed before and after the addition of growing medium, and the mean grey values were calculated using ImageJ to generate curves of adhered growing medium and sandy loam soil.

### Phylogenetic analysis

Representative genomes were downloaded from publicly available databases, sampling each major lineage of land plants. We included intraspecific genomes of *Arabidopsis thaliana* and those of the sister species *A. lyrata*, *A. halleri,* and *A. arenosa* (Supplementary Table S3). Gene families were identified by performing an OrthoFinder2 analysis under default parameters (DIAMOND, fasttree; Emms and Kelly 2019). We identified the gene family containing *ABCG42/3* and performed multiple sequence alignment in MAFFT (−globalpair–maxiterate = 1000; Katoh and Standley 2013). A phylogenetic tree was reconstructed using the best-fitting JTT + C60 + G4 + F model, which accounts for among site rate and compositional heterogeneity, in iQtree with 1000 ultrafast bootstrap replicates (Minh et al. 2020). The consensus tree was rooted using the algal outgroup, *Chlorokybus atmophyticus*.

A parallel analysis focussing on agricultural plant species was performed. Gene families for the species *A. thaliana*, *A. trichopoda*, *B. rapa, G. max, M. truncatula, O. sativa, S. lycopersicum*, *S. tuberosum*, *T. aestivum, T. pratense,* and *Z. mays* were identified using Orthofinder2 as described earlier. The proteins in the orthogroup containing *ABCG43* were aligned using MAFFT v7.48 with an iterative refinement method with WSP and consistency scores (G-INS-I) and visualized in AliView. We repeated gene family reconstruction and the best-fitting model. The consensus tree was rooted using the outgroup *A. trichopoda*, which is sister to all remaining angiosperms.

### Statistics

All statistical analyses were conducted using RStudio, version 1.1453 (R Core Team 2014) and all graphs were generated using the R package ggplot2 (Wickham 2016), with the exception of the heat plot, which was generated using the package matplotlib (Hunter 2007), v3.5.2, in Python, v3.8.

### Root phenotyping

Two sample *t*-tests were conducted using the t.test function to test for differences in the root phenotyping parameter (e.g. mean root hair density) between a candidate line and Col-0. To prevent multiple testing, the alpha level was adjusted to 0.025 or 0.01 using the Bonferroni method.

### Exudate-binding assay

Welch's *t*-tests were conducted using the t.test function to test for differences in the amount of substrate bound to the exudate spots on the nitrocellulose sheet for each candidate line relative to Col-0. A Benjamini–Hochberg adjustment was applied to control for multiple testing, with an alpha level of 0.05.

### RT-PCR analysis

Two sample *t*-tests were conducted using the t.test function to test for differences in the amount of *ABCG43* gene expression (based on the log2 [-dCT] values) for a candidate line relative to Col-0. A Benjamini–Hochberg adjustment was applied to control for multiple testing, with an alpha level of 0.05.

### Root adhesion assay

The analysis of the root-gel adhesion assay used survival analysis as previously described (De Baets et al. 2020; Eldridge et al. 2021). Briefly, Cox PH regression models were conducted using the coxph function within the R survival package. For each Cox PH regression model run, the Wald Statistic (*z*-score) and the hazard ratio with the upper and lower bound confidence intervals are reported. An alpha level of 0.01 was used. Each experiment included >70 biological replicates per genotype and was conducted at least twice.

### Uprooting assay

Linear modelling was conducted using the lm() function to investigate differences in the uprooted compost, uprooted root length and Root Length Density (RLD) between a candidate line relative to Col-0 using R Studio.

### Metabolomics data

Nonmetric multidimensional scaling (NMDS) was conducted using the MetaMDS (using Euclidean distances) in the R vegan package to visually investigate root exudate compositional differences between a candidate line relative to Col-0. In all cases, stress values were below 0.1 and deemed reliable for data interpretation in 2 dimensions.

### Fluorescent signal analysis

Two sample *t*-tests were conducted in the t.test function in R to test for differences in the GFP fluorescent intensity within the same mutant allele background. The “Colocalise” measurement tool in Fiji was to conduct a threshold-based colocalization analysis and generate a Pearson's Correlation Coefficient (PCC) to compare the spatial intensity of the ABCG43-GFP (green) and FM4-64 (red) fluorescence signals in the ABCG43-GFP transgenic lines. A *t*-test was conducted to establish if the PCC significantly differed from 0. The PCC of 6 individual replicates for each line was visualized in R, and the mean and standard error of the PCC were reported.

### Accession numbers

Sequence data from this article can be found in the GenBank/EMBL data libraries under accession numbers_ N75206 (*abcg43-1*) and SALK_201207C (*abcg43-2*).

## Supplementary Material

kiaf193_Supplementary_Data

## Data Availability

The data used in this article will be shared on reasonable request to the corresponding author, Claire Grierson (claire.grierson@bristol.ac.uk).

## Supplementary data

The following materials are available in the online version of this article.

**Supplementary Table S1** Sequence similarity between the Arabidopsis ABCG43 and homologs in crop species.

**Supplementary Table S2** Pearson's correlation coefficient values confirming a high association between ABCG43-GFP and FM4-64 signal in the *abcg43-1:*ABCG43-GFP and *abcg43-2*:ABCG43-GFP lines.

**Supplementary Table S3** Root hair phenotypes in *abcg43* mutants, heterozygote and ABCG43-GFP complemented line are similar to Col-0.

**Supplementary Table S4** The *abcg43* transgenic lines have similar root length densities and total uprooted root length when compared with Col-0.

**Supplementary Table S5** Root length densities and total uprooted root length are similar between the complemented *abcg43* mutant and Col-0.

**Supplementary Table S6** List of primers and associated information used in this study.

**Supplementary Table S7** Primer pairs used to amplify the *ABCG43* coding region and 5′ and 3′ untranslated regions (UTRs) from gDNA,

**Supplementary Figure S1.** GFP-ABCG43 expression in independent transgenic lines.

**Supplementary Figure S2.** Root hair length is not affected in abcg43+/− and ABCG43-GFP complemented lines.

**Supplementary Figure S3.** Root hair density is not altered by ABCG43 expression.

**Supplementary Figure S4.** Comparison of uprooted root length and root length densities of wild-type, *abcg43* mutants, and complemented lines.

**Supplementary Figure S5.** MS/MS spectra in negative ion mode of metabolites upregulated in both *abcg43* mutant alleles compared to Col-0 and elevated fructose levels in *abcg43* mutants.

## Dive Curated Terms

The following phenotypic, genotypic, and functional terms are of significance to the work described in this paper:

seed AmiGo: PO:0009010

root AmiGo: PO:0009005
