# Supplementary material for: A highly conserved ABCG transporter mediates root–soil cohesion in Arabidopsis
Source: Plant Physiol. 2025 May 12;198(1):kiaf193. doi: 10.1093/plphys/kiaf193 (PMC12084803; doi:10.1093/plphys/kiaf193)
Supplement: kiaf193_Supplementary_Data [file kiaf193_supplementary_data.pdf]

***Plant Physiology* Supplemental Materials**

Article title: **A highly conserved ABCG transporter mediates root-soil cohesion in Arabidopsis**

Authors: Bethany M Eldridge, Emily R Larson, Lucy Mahony, James Clark, Jumana Akhtar, Clarice Noleto-Dias, Jane L Ward, Claire S Grierson

Table S1 Sequence similarity between the Arabidopsis ABCG43 and homologs in crop species.

Protein	Similarity to AtABCG43 percent identity	Similarity to AtABCG43 e value
A. tricopodium ATR0787G017	58.097	0.0
A. tricopodium ATR0787G223	55.991	0.0
T. pratense TPR.T12966	56.004	0.0
M. truncatula MTR_4g123850	60.057	0.0
G. max Glyma_17G039200	60.088	0.0
G. max Glyma_07G233900	59.236	0.0
G. max Glyma_17G039300	61.827	0.0
B. rapa Bra037088	72.02	0.0
A. thaliana ABCG30	71.079	0.0
A. thaliana ABCG41	72.806	0.0
A. thaliana ABCG43	100.00	0.0
A. thaliana ABCG42	94.816	0.0
B. rapa Bra012797	82.092	0.0
S. lycopersicum Solyc01g101070	60.920	0.0
S. tuberosum PGSC0003DMT400046986	59.865	0.0
B. rapa Bra003137	56.044	0.0
A. thaliana ABCG37	57.872	0.0
A. thaliana ABCG33	57.436	0.0
B. rapa Bra005208	57.895	0.0
B. rapa Bra017198	58.458	0.0
O. sativa Os12t0512700-02	53.558	0.0
T. aestivum TraesCS7D01G080300	54.311	0.0
T. aestivum TraesCS7A01G085800	55.172	3.81e-148
T. aestivum TraesCS4A01G384800	54.09	0.0
Z. mays Zm00001eb406530_T001	56.312	0.0
T. aestivum TraesCS4B01G054700	55.103	0.0
T. aestivum TraesCS5B03G0424400	54.462	0.0
T. aestivum TraesCS5D02G163700	56.046	0.0
T. aestivum TraesCS7B01G381200	53.314	0.0
T. aestivum TraesCS7B02G381000	54.422	0.0
T. aestivum TraesCS7D01G465900	54.919	0.0
T. aestivum TraesCS7A02G478500	54.81	0.0
T. aestivum TraesCS5D01G163600	54.889	0.0
T. aestivum TraesCS5A01G158300	55.318	0.0
T. aestivum TraesCS5B01G155600	51.42	0.0
T. aestivum TraesCS5D02G163500	55.974	0.0
T. aestivum TraesCS5A02G158100	55.263	0.0

Percentage identity and e values reported from BLASTP.

Table S2 Pearson's correlation coefficient values confirming a high association between ABCG43-GFP and FM4-64 signal in the *abcg43-1:*ABCG43-GFP and *abcg43-2*:ABCG43-GFP lines.

***abcg43-1*:ABCG43-GFP**			***abcg43-2*:ABCG43-GFP**		
Replicate	***Pearson's Correlation Coefficient***	***P-value (t-test)***	***Replicate***	***Pearson's Correlation Coefficient***	***P-value (t-test)***
1	0.789	<0.0001	1	0.944	<0.0001
2	0.880	<0.0001	2	0.910	<0.0001
3	0.881	<0.0001	3	1.000	<0.0001
4	0.928	<0.0001	4	0.967	<0.0001
5	0.900	<0.0001	5	1.000	<0.0001
6	0.950	<0.0001	6	1.000	<0.0001
Mean	0.888	<0.0001	Mean	0.970	<0.0001
Standard error	0.023	/	Standard error	0.015	/

For each transgenic line, a Pearson's correlation coefficient (with corresponding P-values) quantifying the signal intensity overlap of the GFP (green) and FM4-64 (red) channels was calculated for 6 individual replicates (microscopy images) in Fiji/ImageJ.

Table S3 Root hair phenotypes in *abcg43* mutants, heterozygote and ABCG43-GFP complemented line are similar to Col-0.

Line	***Root hair density (per mm length of root)***		***Root hair length (mm)***	
Col-0	41.500 ± 1.579<br>*n* = 10	-	0.499 ± 0.008<br>n = 10	-
abcg43-1-/-	41.000 ± 1.693<br>n = 10<br>**No difference**	t = -0.210<br>P > 0.05<br>d.f. = 18	0.491 ± 0.007<br>n = 10<br>**No difference**	t = -0.003<br>P > 0.05<br>d.f. = 18
abcg43-2-/-	41.500 ± 1.753<br>n = 10<br>**No difference**	t = 0.000<br>P > 0.05<br>d.f. = 18	0.499 ± 0.007<br>n = 10<br>**No difference**	t = -0.003<br>P > 0.05<br>d.f. = 18
Col-0	39.700 ± 1.265<br>*n* = 10	-	0.500 ± 0.007<br>*n* = 10	-
abcg43-1+/-	40.000 ± 1.238<br>*n* = 10<br>**No difference**	t = 0.164<br>P > 0.05<br>*d.f.* = 18	0.500 ± 0.007<br>*n* = 10<br>**No difference**	t = -0.009<br>P > 0.05<br>*d.f.* = 18
Col-0	41.733 ± 0.742<br>*n* = 10	-	0.466 ± 0.003<br>*n* = 10	-
abcg43-2+/-	42.866 ± 0.686<br>*n* = 10<br>**No difference**	t = 0.069<br>P > 0.05<br>*d.f.* = 18	0.434 ± 0.003<br>*n* = 10<br>**No difference**	t = -1.121<br>P > 0.05<br>*d.f.* = 18.
Col-0	41.200 ± 1.191<br>*n* = 10	-	0.485 ± 0.005<br>*n* = 10	-
abcg43-1:ABCG43-GFP	41.200 ± 1.405<br>*n* = 10<br>**No difference**	t = 0.000<br>P > 0.05<br>*d.f.* = 18	0.482 ± 0.006<br>*n* = 10<br>**No difference**	t = -0.126<br>P > 0.05<br>*d.f.* = 18
Col-0	40.2667 ± 1.300<br>*n* = 10	-	0.428 ± 0.004<br>*n* = 10	-
abcg43-2:ABCG43-GFP	32.800 ± 1.340<br>*n* = 10<br>**No difference**	t = 0.156<br>P > 0.05<br>*d.f.* = 18	0.428 ± 0.004<br>*n* = 10<br>**No difference**	t = -1.109<br>P > 0.05<br>*d.f.* = 18

Thick lines separate data that were collected and analyzed using the Col-0 within the same experiment. The *n* number indicates the number of individual seedlings measured.

Table S4 The *abcg43* transgenic lines have similar root length densities and total uprooted root length when compared with Col-0.

Line	Difference between RLD (km m^{-3})		Difference between total uprooted length (cm)	
abcg43-1	No difference<br>*n* = 5	t = 0.57<br>$P > 0.05^{ns}$<br>*d.f.* = 8	No difference<br>*n* = 10	t = -0.68<br>$P > 0.05^{ns}$<br>*d.f.* = 18
abcg43-2	No difference<br>*n* = 5	t = 0.26<br>$P > 0.05^{ns}$<br>*d.f.* = 8	No difference<br>*n* = 10	t = -0.18<br>$P > 0.05^{ns}$<br>*d.f.* = 18
abcg43-2: ABCG43-GFP	No difference<br>*n* = 5	t = -0.11<br>$P > 0.05^{ns}$<br>*d.f.* = 8	No difference<br>*n* = 10	t = 1.87<br>$P > 0.05^{ns}$<br>*d.f.* = 18

Mean difference and output of univariate linear model (t-test) are shown for each candidate line relative to Col-0.

Table S5 Root length densities and total uprooted root length are similar between the complemented *abcg43* mutant and Col-0.

**Line**	**Mean estimated pot root length (km)**	**Difference between RLD (km m^{-3})**		**Mean uprooted root length (cm)**	**Difference between total uprooted length (cm)**	
Col-0	0.0150 (0.0005)	-	-	108.1176<br>(5.37)	-	-
abcg43-2: ABCG43-GFP	0.02093497	**No difference**<br>***n* = 5**	t = -0.11<br>$P > 0.05^{ns}$<br>*d.f.* = 8	107.641<br>(25.70)	**No difference**<br>***n* = 10**	t = 1.87<br>$P > 0.05^{ns}$<br>*d.f.* = 18

Mean root lengths from representative experiments are provided with standard error in parentheses. Mean difference and output of univariate linear model (t-test) are shown for each candidate line relative to Col-0. The results are representative of two replicate experiments, each containing at least 10 individuals. The *n* number indicates the number of individual seedlings measured.

Table S6 List of primers and associated information used in this study.

qRT-PCR primer probes

Gene target	Probe	sequence	Fluorophore	Detection (nm)	Quencher
***ABCG43* (At4g15236)**	ABCG43_probe	AGGCCTTATGATGCCACTGACACC	6-FAM	518	BHQ-1
**Galactose oxidase/kelch repeat superfamily protein (At5g15710)**	At5g15710F-box_probe	TTTCTCGTGGGAAGGATTGGCCTC	HEX	553	BHQ-1
**UBC21 (Ubiquitin-conjugating enzyme 21) (At5g25760)**	UBC21_probe	GGAGTCCTGCTTGGACGCTTCAGTCTG	Cy5	667	BHQ-3

RT-PCR primers

Gene target	Primer	Sequence (5'-3')	Hybridization location	cDNA product (bp)	gDNA product (bp)
***ABCG43* (At4g15236)**	*ABCG43_cDNA_F*	AAATGGGGATGGTGATCAGGT	*ABCG43* exon 1	879	1464
	ABCG43_cDNA_R	ATATTCCTGGTCTCGAAGCATCT	*ABCG43* exon 8		
Elongation Factor 1α	*EF-1a*_F	TTGGGTGGTATTGACAAGCG	*EF-1a* exon 1	566	659
	*EF-1a*_R	GCCTCAAGGAGAGTTGGTC	*EF-1a* exon 2		

Gateway cloning primers

Gene target	Primer	Sequence (5'-3')	Hybridization localization	Product size (bp)
***ABCG43* (At4g15236)**	*ABCG43_att*B_F	GGGGACAAGTTTGTACAAAAAAGCAGGCTCTATGACAATGCCTCAAACAGATGG	Exon 1	6048
***ABCG43* (At4g15236)**	*ABCG43_att*B_R	GGGGACCACTTTGTACAAGAAAGCTGGGTGCTTCTTTTGGAAATTGAGTTTACC	Exon 23	6048

Two-step qRT-PCR TaqMan assay, using specific fluorophores with Black Hole Quenchers. '_F' or '_R' refer to the forward and reverse gene-specific primers and the Gateway *att* site sequences are underlined.

Table S7 Primer pairs used to amplify the *ABCG43* coding region and 5' and 3' untranslated regions (UTRs) from gDNA.

Primer name	***5' – 3' sequence (nt)***	***Hybridization location***	***Product size (bp)***
*ABCG43*_amplicon1_F	CCAGTCACGACGTTGTAAAACGACG<br>GCCAGTGCAACATGCTTGTGAGGAG	5' UTR	1729
*ABCG43*_amplicon1_R	CTGATCACCATCCCCATTTTCAAG	Exon 1	
*ABCG43*_amplicon2_F	CTTCGTTATAAAGAACAATCCG	Exon 1	2085
*ABCG43*_amplicon2_R	GCAATATGGTTTGTCTCTGTG	Exon 10	
*ABCG43*_amplicon3_F	GTCCACAGAGGAAATCTGTTGC	Exon 9	2130
*ABCG43*_amplicon3_R	CTCAGACAAGGAAGCTAAAGGAAGG	Exon 16	
*ABCG43*_amplicon4_F	CTTGACGTTCTCTCAGGAAGG	Exon 16	2630
*ABCG43*_amplicon4_R	TTGTGAGCGGATAACAATTTCACAC<br>AGGAAAAGCTTGTTGTTGTTCACCG	3' UTR	

'_F' or '_R' refer to the forward and reverse primers.

Supplemental Figures

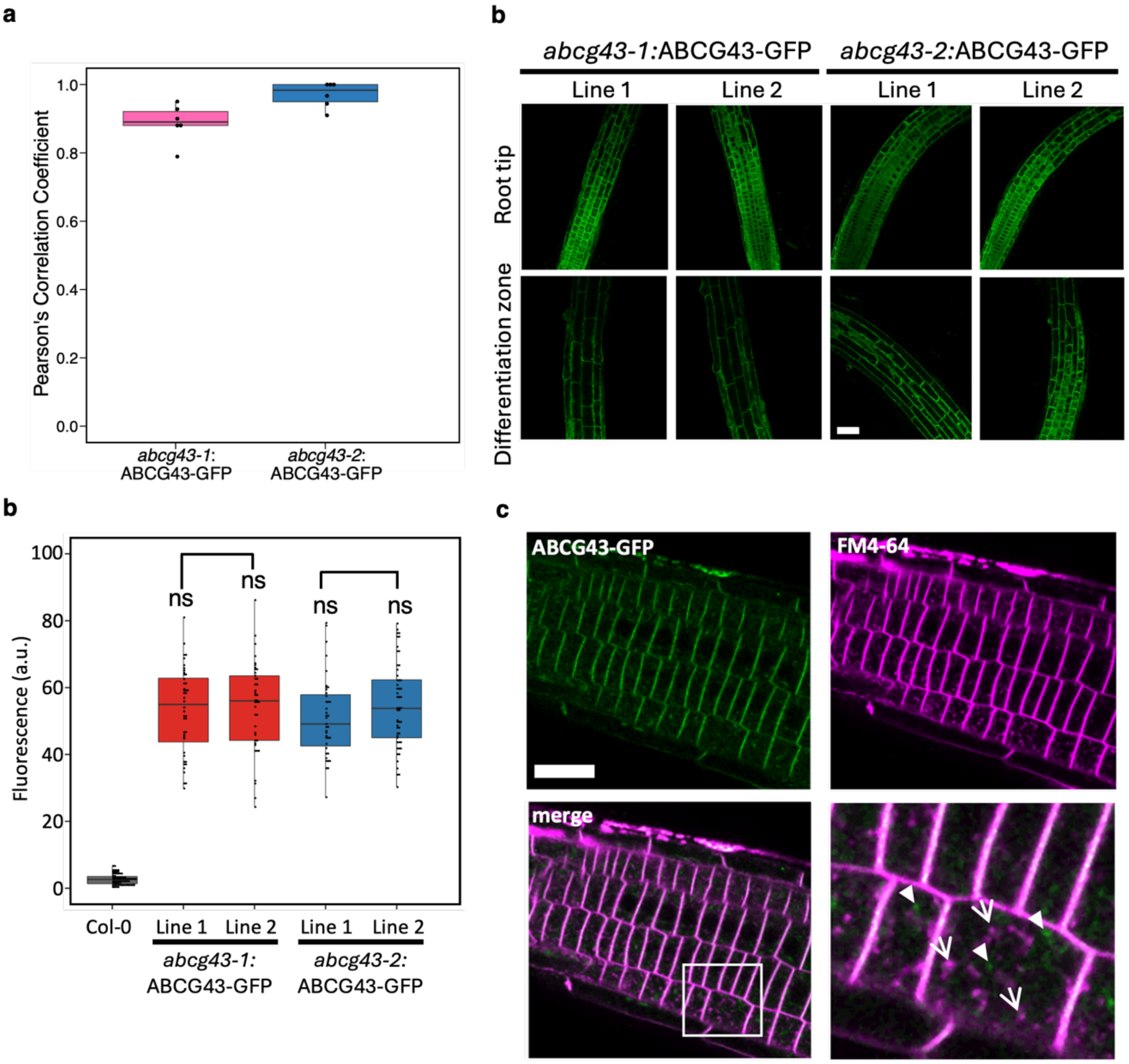

Fig S1. GFP-ABCG43 expression in independent transgenic lines.
(a) Pearson's correlation coefficient values confirming a high association between the distribution of ABCG43-GFP and FM4-64 at the plasma membrane in the *abcg43-1:*ABCG43-GFP and *abcg43-2*:ABCG43-GFP lines. For each line, a Pearson's correlation coefficient quantifying the signal intensity overlap of the GFP (green) and FM4-64 (red) channels was calculated for 6 individual replicates (microscopy images) in Fiji/ImageJ. **(b)** ABCG43-GFP fluorescence in *abcg43-1* and *abcg43-2* lines showing similar fluorescence intensity in roots. **(c)** A graphical representation of the fluorescence intensities in the independent transgenic lines shown in **(b).** Scale bars = 50 μm. Linear models: no significant difference between abcg43-1 Line 1 and Line 2 ($F = 2.23$, d.f. = 82, $P > 0.05$) and abcg43-2 Line 1 and Line 2 ($F = 2.01$, d.f. = 89, $P > 0.05$)). **(d)** Representative ABCG43-GFP stable line co-labelled with FM4-64 showing co-localisation at the plasma membrane. Lower right image is a magnification of the merge image within the white box. Arrowheads highlight ABCG43-GFP compartments and arrows highlights FM4-64 compartments.

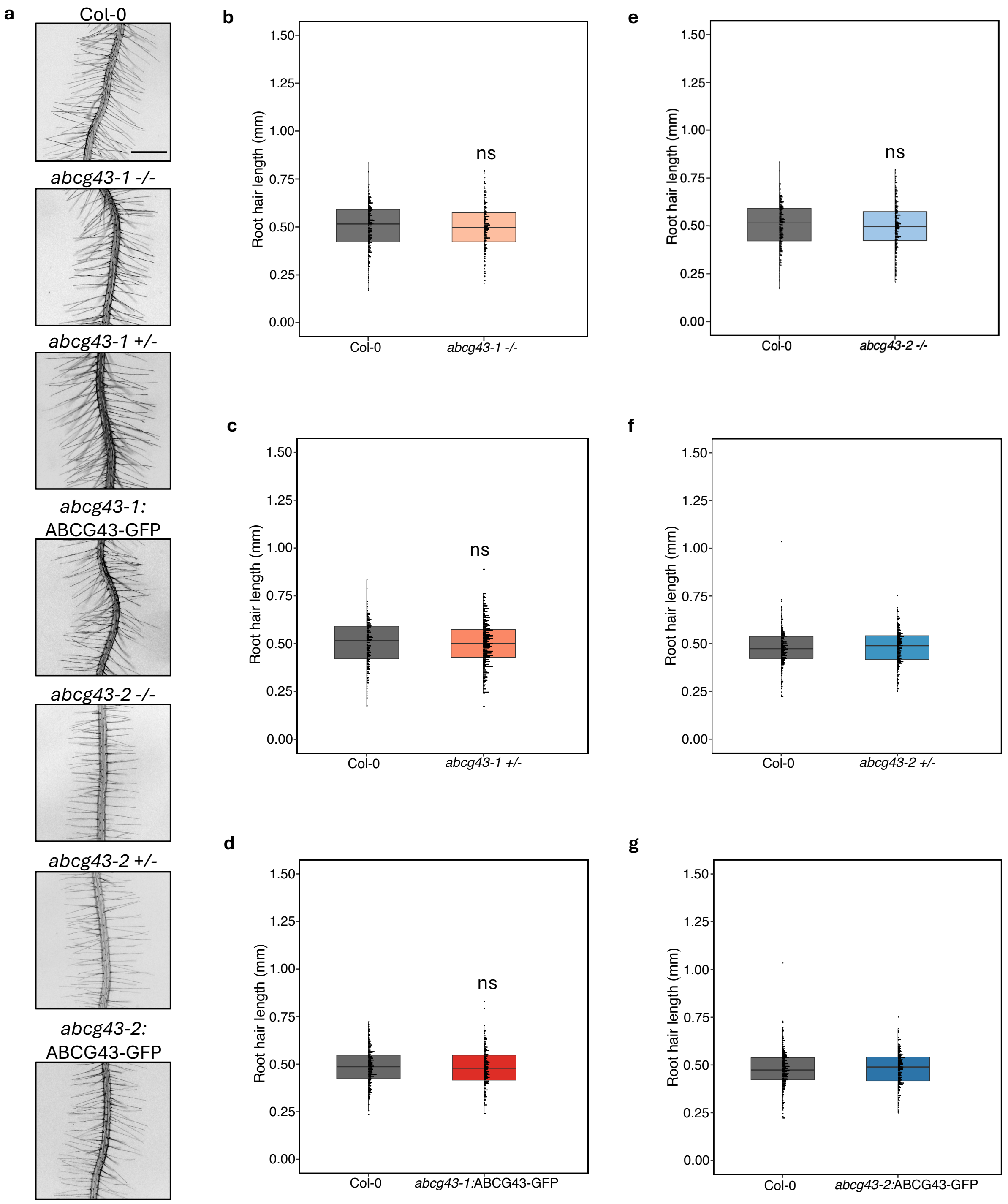

Fig. S2 Root hair length is not affected in *abcg43*+/- and ABCG43-GFP complemented lines. **(a)** Representative images of root hairs in the wild type, *abcg43* heterozygous, and complemented lines and their **(b-g)** quantification. The root hairs in the transgenic lines were not different from the wild type. Scale bar = 1 mm (t-tests, no significant difference in all cases – see Table S3).

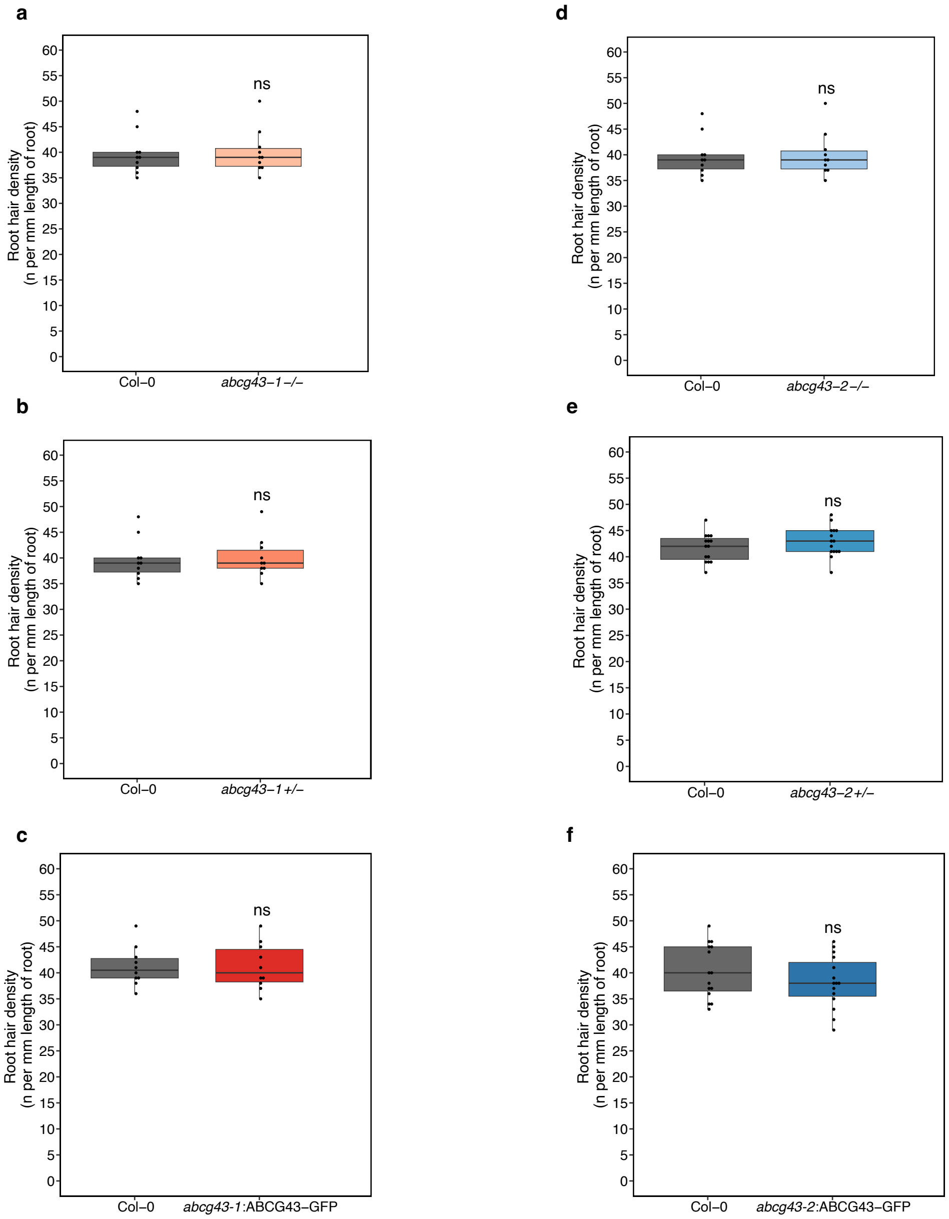

Fig. S3 Root hair density is not altered by *ABCG43* expression. Root length density (RLD) was measured in **(a, d)** *abcg43* and **(c, e)** complemented lines compared to wild type and showed no statistical difference in 3-week-old plants grown in compost (t-tests, no significant difference in all cases – see Table S3).

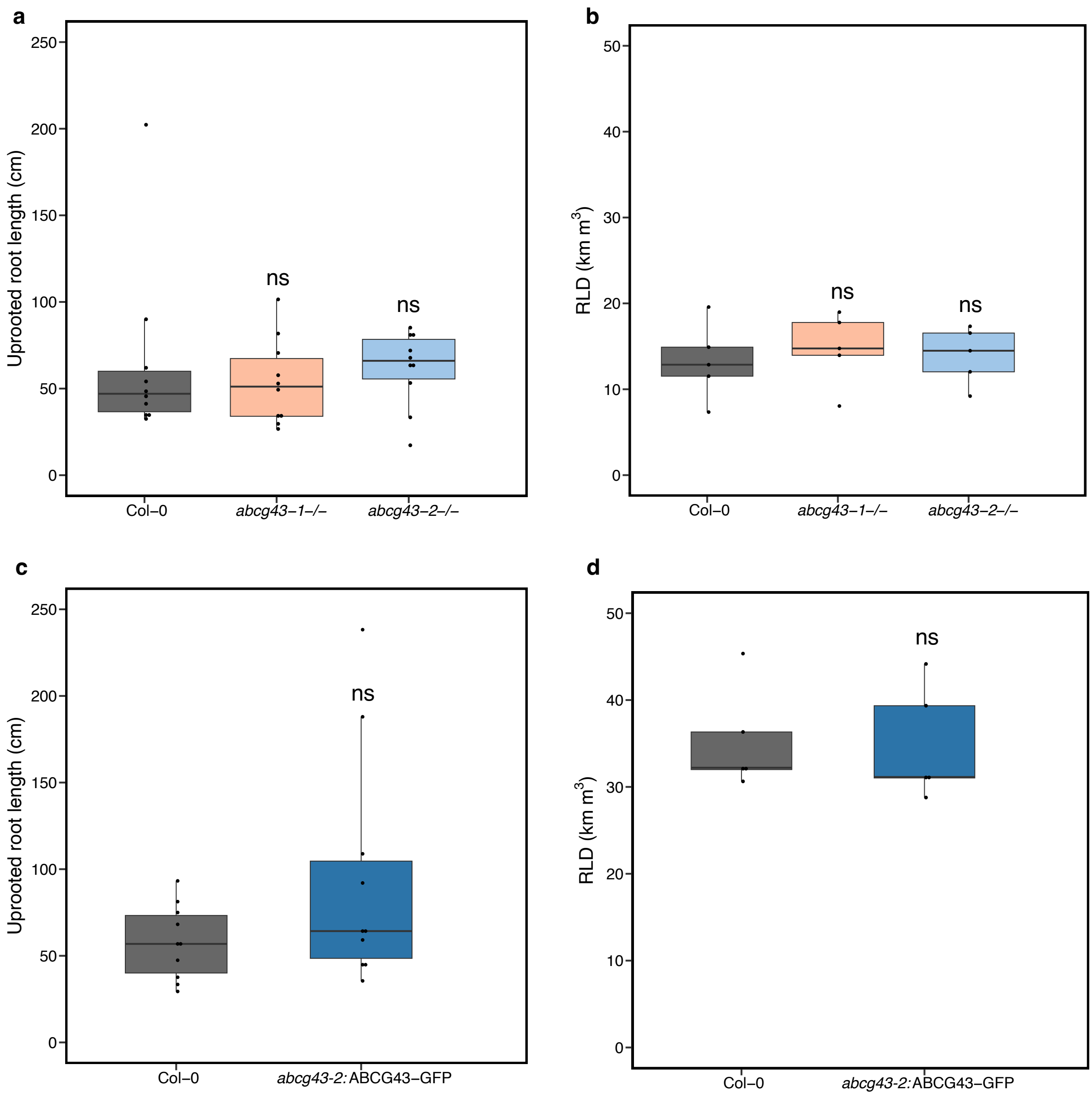

Fig. S4. Comparison of uprooted root length and root length densities of wild-type, *abcg43* mutants, and complemented lines. The length of the roots after uprooting were not different between wild-type and **(a)** *abcg43* mutants or **(b)** the complemented line (t-tests, no significant differences in all cases – see Table S3). The root length densities (RLD) of the **(c)** *abcg43* mutants and **(d)** complemented line were also not different than wild type. (n = 10-15 4-week-old plants grown in compost; t-tests, no significant differences in all cases – see Tables S4 and S5).

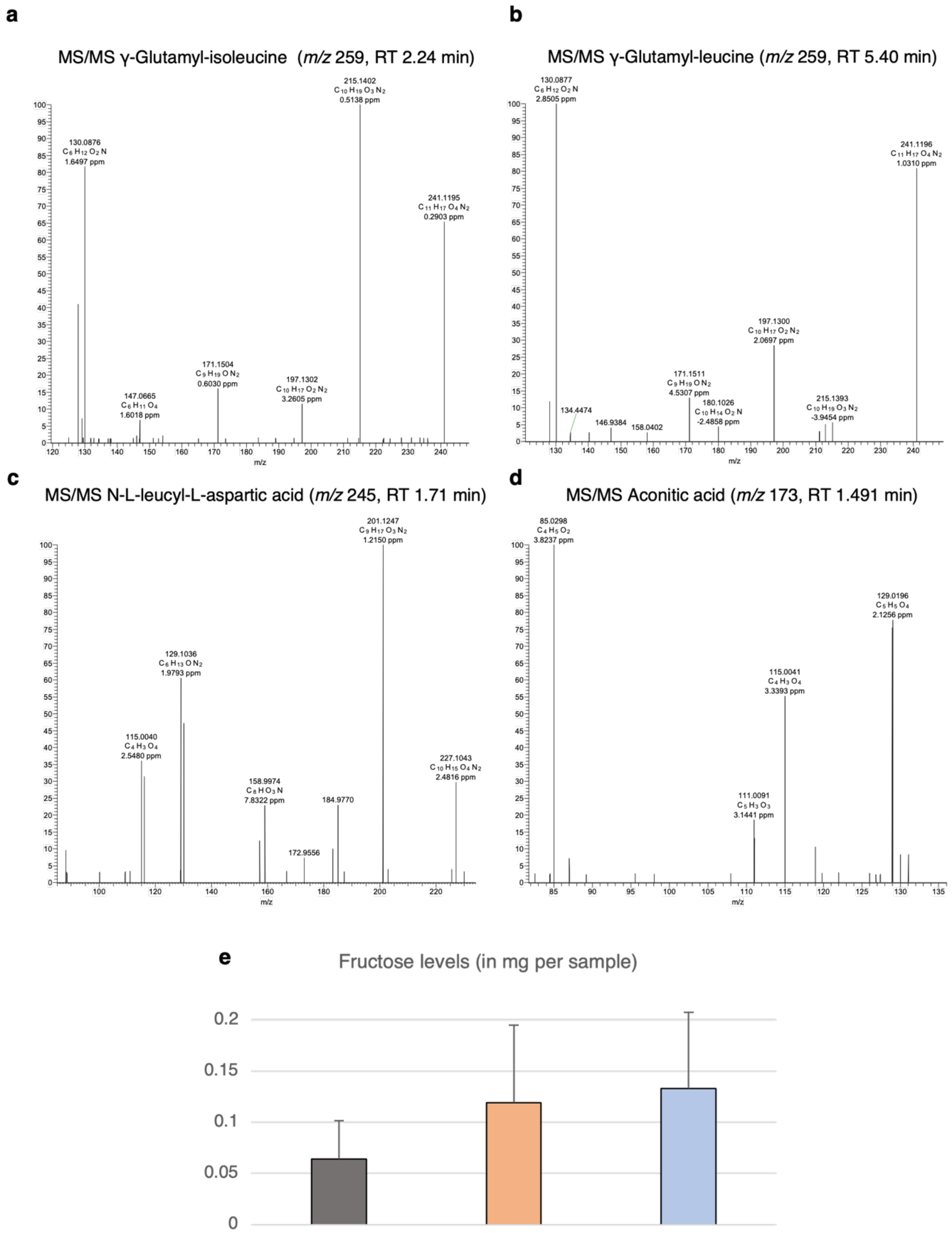

Fig. S5. MS/MS spectra in negative ion mode of metabolites upregulated in both *abcg43* mutant alleles compared to Col-0 and elevated fructose levels in *abcg43* mutants. (a) γ-glutamyl-leucine, **(b)** N-L-leucyl-L-aspartic acid, and **(c)** the organic acid aconitic acid **(d). (e)** Absolute quantitation via ^{1}H-NMR integration of fructose-specific resonances against internal standard 3-(trimethylsilyl) propionic acid-d_4 (TSP-d_4, 0.01% w/v).
